# Lrp Family Regulator SCAB_Lrp2 Responds to the Precursor Tryptophan and Represses the Thaxtomin Biosynthesis in *Streptomyces scabies*

**DOI:** 10.1111/mpp.70036

**Published:** 2024-12-01

**Authors:** Haoyang He, Lijuan Tang, Mingrui Song, Hui Chen, Youquan Zou, Xueyan Li, Endong Yang, Hang Wu, Buchang Zhang, Jing Liu

**Affiliations:** ^1^ School of Life Sciences Anhui Agricultural University Hefei China; ^2^ School of Life Sciences, Institute of Physical Science and Information Technology Anhui University Hefei China

**Keywords:** cluster‐situated regulator, Lrp regulator, pathogenicity, *Streptomyces scabies*, thaxtomin, tryptophan

## Abstract

*Streptomyces scabies* is a well‐researched plant pathogen belonging to the genus *Streptomyces*. Its virulence is linked to the production of the secondary metabolite thaxtomin A, which is tightly regulated at the transcriptional level. The leucine‐responsive regulatory protein (Lrp) family is conserved in prokaryotes and is involved in various crucial biological processes. However, the regulatory interaction between Lrp protein and pathogenic *Streptomyces* species remains poorly understood. This study aims to explore the role of SCAB_Lrp2 in regulating thaxtomin biosynthesis and pathogenicity, and to analyse the shared and unique features of Lrp homologues in *S. scabies*. We observed that SCAB_Lrp2 (SCAB_75421) showed significant homology with SCAB_Lrp, a recognised activator of thaxtomin A production in *S. scabies*. Our results revealed a regulatory interaction between SCAB_Lrp2 and SCAB_Lrp in terms of their targets, although SCAB_Lrp2 does not respond to the amino acid‐effectors of SCAB_Lrp. In contrast to SCAB_Lrp, deletion of *SCAB_Lrp2* resulted in a notable increase in thaxtomin A production with the emergence of a hypervirulent phenotype in *S. scabies*. Further analysis revealed that SCAB_Lrp2 represses the transcription of the thaxtomin biosynthetic gene cluster by directly regulating the cluster‐situated regulator (CSR) gene *txtR*. Moreover, the precursor of thaxtomin, tryptophan, acts as an effector of SCAB_Lrp2, strengthening the repressive effect on thaxtomin biosynthesis through *txtR*. These findings provide new insights into the functional conservation and diversity of Lrp homologues involved in the biosynthesis of thaxtomin phytotoxins in pathogenic *Streptomyces* species.

## Graphical abstract

Lrp family regulator SCAB_Lrp2 from pathogenic *Streptomyces scabies* responds to the precursor tryptophan and represses thaxtomin biosynthesis.

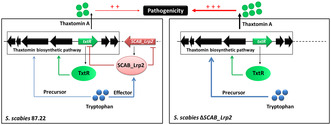

## 1 Introduction

Gram‐positive *Streptomyces* species are found extensively in soil and display a complex life cycle. Their metabolites are widely used in medicine, health, animal husbandry, and as agricultural herbicides. Within the diverse *Streptomyces* genus, some strains have been identified as plant pathogens, with *S. scabies*, *S. turgidiscabies* and *S. acidiscabies* being the most studied (Loria, Kers, and Joshi 2006; Lerat, Simao‐Beaunoir, and Beaulieu 2009). These pathogens cause scab lesions, either raised or sunken, on economically important crops, such as potato, turnip, beet, peanut and sweet potato. Thaxtomin A is the primary determinant of pathogenicity in *S. scabies* and related species (Loria et al. 2008). Thaxtomin A, a member of the nitrated diketopiperazines family, acts as an inhibitor of plant cellulose biosynthesis, though its specific target and molecular mechanism remain unknown (Bischoff et al. 2009). Moreover, thaxtomin A exhibits significant inhibition of seedling growth and causes abnormal cell enlargement in plants even at extremely low concentrations (Bischoff et al. 2009), suggesting its potential as a natural bioherbicide (King and Calhoun 2009; Wang et al. 2020).

The *txt* gene cluster, responsible for thaxtomin biosynthesis, consists of seven genes and is highly conserved among *Streptomyces* species that cause scab formation (Huguet‐Tapia et al. 2016). These genes are organised within a segment of approximately 18.3 kb (Li et al. 2019; Huguet‐Tapia et al. 2016). Six of these genes (*txtA*, *txtB*, *txtC*, *txtD*, *txtE* and *txtH*) encode enzymes essential for thaxtomin biosynthesis, while *txtR* encodes the cluster‐situated regulator (CSR), which activates thaxtomin biosynthesis (Joshi et al. 2007; Yang, Tauschek, and Robins‐Browne 2011). Besides TxtR, thaxtomin A production is tightly regulated by two pleiotropic regulators: CebR (Francis et al. 2015) and SCAB_Lrp (Liu et al. 2023). CebR acts as a repressor, while SCAB_Lrp functions as an activator of thaxtomin A production in *S. scabies* (Francis et al. 2015; Liu et al. 2023).

The leucine‐responsive regulatory protein (Lrp) family, widely found in prokaryotes, plays key roles in various biological processes (Ziegler and Freddolino 2021). Lrp proteins regulate target genes by binding to promoters through their N‐terminal helix‐turn‐helix motif, while the regulation is modulated by an effector‐binding domain at their C‐terminus (Ziegler and Freddolino 2021). However, the regulatory role of Lrp proteins in thaxtomin A production and pathogenicity in *S. scabies* remains unclear.

Our laboratory has recently made significant progress in understanding the regulatory role of SCAB_Lrp in thaxtomin synthesis in *S. scabies* (Liu et al. 2023). Sequence alignment revealed that SCAB_75421 exhibited high similarity to SCAB_Lrp, suggesting that it may also function as a regulator, now referred to as SCAB_Lrp2. Therefore, this study aims to dissect the regulatory mechanisms of SCAB_Lrp2 in controlling thaxtomin biosynthesis and pathogenicity, and to analyse the shared and unique features of Lrp homologues in *S. scabies*.

## 2 Results

### 2.1 Lrp Homologue SCAB_Lrp2 Responds to Tryptophan in *S. scabies*

**FIGURE 1:**
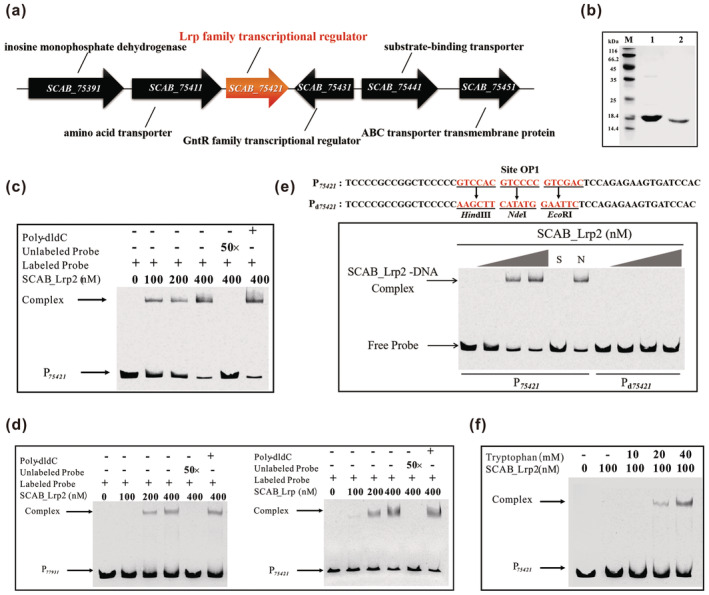
Lrp homologue SCAB_Lrp2 responds to tryptophan in *Streptomyces scabies* 87.22. (a) Genetic locus organization of the SCAB_Lrp2. (b) SDS‐PAGE analysis of purified SCAB_Lrp2. Lanes: M, 116‐kDa protein ladder; 1, His_6_‐tagged SCAB_Lrp2; 2, untagged SCAB_Lrp2. (c) Electrophoretic mobility shift assays (EMSAs) of binding affinity of SCAB_Lrp2 to probe P_*75421*_. P_*75421*_ represents the promoter region of *SCAB_Lrp2*. (d) EMSAs of binding affinity of SCAB_Lrp2 to probe P_*77931*_ and SCAB_Lrp to probe P_*75421*_. P_*77931*_ represents the promoter region of *SCAB_Lrp*. (e) EMSAs of binding affinity of SCAB_Lrp2 to probes P_*75421*_ and P_d*75421*_. HindIII, NdeI and EcoRI sites were introduced into the site OP1 in P_*75421*_ to generate mutated probe P_d*75421*_. Underlined text indicates altered nucleotides. The amounts of SCAB_Lrp2 used were 0, 100, 200 and 400 nM. S: 400 nM SCAB_Lrp2 with unlabelled specific probe (50‐fold); N: 400 nM SCAB_Lrp2 with nonspecific probe poly‐dIdC (50‐fold). (f) EMSAs of binding affinity of SCAB_Lrp2 to probe P_*75421*_ in presence of tryptophan.

SCAB_75421, a 150‐amino acid homologue of the Lrp family (Figure 1a), was predicted from the genomic sequence of *S. scabies* 87.22. With 32% identity to our previously reported SCAB_Lrp (Liu et al. 2023), it is now referred to as SCAB_Lrp2. A comparison of the amino acid sequences of SCAB_Lrp2 and SCAB_Lrp revealed that the N‐terminal DNA‐binding regions showed greater conservation, while the C‐terminal effector‐binding regions displayed more variability (Figure S1).

Recombinant His_6_‐tagged SCAB_Lrp2 was produced in *Escherichia coli* BL21 (DE3) and subsequently purified by removing the His_6_‐tag using a thrombin cleavage kit (Abcam). (Figure 1b). To determine whether SCAB_Lrp2 directly regulates its own gene, electrophoretic mobility shift assays (EMSAs) were performed with SCAB_Lrp2 binding to its own promoter (probe P_*75421*_). The results showed a clearly shifted band when SCAB_Lrp2 was incubated with probe P_*75421*_, confirming its direct interaction with its own promoter (Figure 1c), similar to SCAB_Lrp. Additionally, we performed EMSAs with SCAB_Lrp2 binding to the SCAB_Lrp promoter (probe P_*77931*_) or conversely. The results showed that SCAB_Lrp2 and SCAB_Lrp could bind to each other's promoters, forming SCAB_Lrp2‐P_*77931*_ and SCAB_Lrp‐P_*75421*_ complexes, albeit with slight differences in affinity (Figure 1d). The DNA‐binding site of SCAB_Lrp (site OP) was previously identified (Liu et al. 2023) and served as a basis for identifying SCAB_Lrp2's binding site. Through BLAST analysis and EMSAs, an 18 bp sequence (5′‐GTCCACGTCCCCGTCGAC‐3′), located within the SCAB_Lrp2 promoter region (referred to as site OP1), was identified as the specific DNA‐binding site of SCAB_Lrp2. This was confirmed by using a mutated probe P_d*75421*_ (Figure 1e).

To explore SCAB_Lrp2's response to the effector amino acids of SCAB_Lrp (methionine and phenylalanine), EMSAs were performed with SCAB_Lrp2 and probe P_*75421*_ in the presence of these amino acids. Neither methionine nor phenylalanine affected the binding affinity of SCAB_Lrp2 for probe P_75421_ (Figure S2). To identify the potential effectors of SCAB_Lrp2, EMSAs were conducted with other amino acids (Figure S3). The results showed that only tryptophan enhanced the binding affinity of SCAB_Lrp2 for probe P_*75421*_ (Figure 1f), indicating that tryptophan acts as a specific effector of SCAB_Lrp2.

In summary, these findings highlight the regulatory interaction between SCAB_Lrp2 and SCAB_Lrp concerning their target promoters. However, SCAB_Lrp2 does not respond to the amino acid effectors of SCAB_Lrp, showcasing both the functional conservation and diversity of these two Lrp proteins.

### 2.2 Deletion of *SCAB_Lrp2* Enhances the Production of Thaxtomin A

**FIGURE 2:**
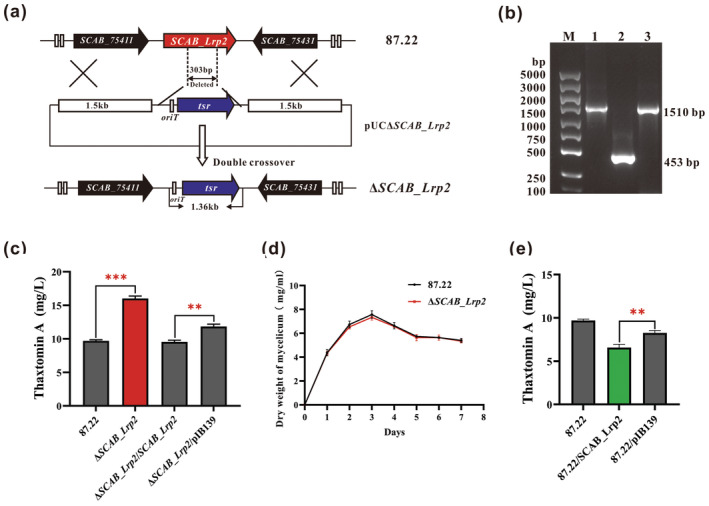
Deletion of *SCAB_Lrp2* enhances thaxtomin A production. (a) Schematic deletion of *SCAB_Lrp2* by homologous recombination in *Streptomyces scabies* 87.22. By the homologous chromosomic recombination, a 303‐bp fragment within the *SCAB_Lrp2* gene was replaced by the *tsr* gene. (b) PCR confirmation of mutant Δ*SCAB_Lrp2* using primers 75421‐CF/R. Lanes: M, 5000‐bp DNA ladder; 1, the positive control, 1510 bp amplified from pUCΔ*SCAB_Lrp2*; 2, the negative control, 453 bp amplified from 87.22; 3, the sample, 1510 bp amplified from Δ*SCAB_Lrp2*. (c) Thaxtomin A production in *S. scabies* 87.22, Δ*SCAB_Lrp2*, Δ*SCAB_Lrp2*/*SCAB_Lrp2* and Δ*SCAB_Lrp2*/pIB139 cultured for 7 days through HPLC analysis. Mean values of three replicates are shown, with the standard deviation indicated by error bars (Student's *t* test; **p < 0.01, ***p < 0.001). (d) Growth curves of *S. scabies* 87.22 and Δ*SCAB_Lrp2*. The two strains were cultured in oat bran broth (OBB), and the dry weights of mycelia were measured. Mean values of three replicates are shown, with the standard deviation indicated by error bars. (e) Thaxtomin A production in *S. scabies* 87.22 87.22/*SCAB_Lrp2*, and 87.22/pIB139. Mean values of three replicates are shown, with the standard deviation indicated by error bars (Student's *t* test; **p < 0.01).

To investigate the influence of SCAB_Lrp2 on thaxtomin A production, the *SCAB_Lrp2* gene in *S. scabies* 87.22 was disrupted by thiostrepton resistance gene (*tsr*) replacement through homologous chromosomal recombination (Figure 2a). PCR analysis confirmed the successful generation of the mutant strain Δ*SCAB_Lrp2* (Figure 2b). When cultured in oat bran broth (OBB), thaxtomin A production in Δ*SCAB_Lrp2* increased by 62.8% (*p* < 0.001) compared to the parent strain 87.22, as determined by fermentation and HPLC analysis (Figure 2c). Thaxtomin A production was restored to the wild‐type level in the complemented strain Δ*SCAB_Lrp2*/*SCAB_Lrp2* (Figure 2c). SCAB_Lrp2 did not affect cell growth of *S. scabies*, as indicated by similar biomass curves in terms of mycelial dry weight between Δ*SCAB_Lrp2* and the parent strain 87.22 (Figure 2d).

To confirm the regulatory role of SCAB_Lrp2 in thaxtomin A production, pIB*SCAB_Lrp2* (Table S1) and pIB139 were introduced into 87.22. Thaxtomin A production in 87.22/*SCAB_Lrp2* decreased by 25.7% (*p* < 0.01) compared to 87.22/pIB139 (Figure 2e). Overall, these results showed that SCAB_Lrp2 negatively regulates thaxtomin A production without affecting *S. scabies* growth.

### 2.3 SCAB_Lrp2 Directly Represses the Transcription of the CSR Gene *txtR*

**FIGURE 3:**
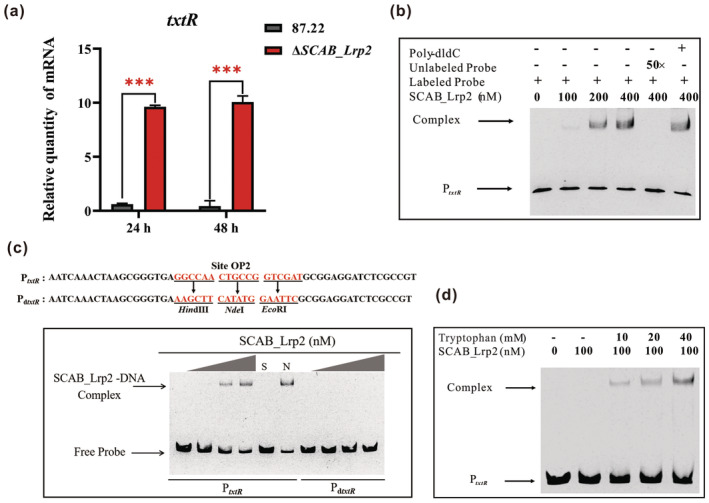
SCAB_Lrp2 directly represses the transcription of the CSR gene *txtR*. (a) Effects of *SCAB_Lrp2* deletion on transcriptional levels of *txtR*. Reverse transcription‐quantitative PCR was used to quantify the amounts of transcripts in 87.22 and Δ*SCAB_Lrp2* cultured for 24 and 48 h in oat bran broth (OBB). Mean values of three replicates are shown, with the standard deviation indicated by error bars (Student's *t* test; ***p < 0.001). (b) Electrophoretic mobility shift assays (EMSAs) of binding affinity of SCAB_Lrp2 to probe P_*txtR*_. P_*txtR*_ represents the promoter region of *txtR*. (c) EMSAs of binding affinity of SCAB_Lrp2 to probes P_*txtR*_ and P_d*txtR*_. HindIII, NdeI and EcoRI sites were introduced into the site OP2 in P_*txtR*_ to generate mutated probe P_d*txtR*_. Underlined text indicates altered nucleotides. The amounts of SCAB_Lrp2 used were 0, 100, 200 and 400 nM. S: 400 nM SCAB_Lrp2 with unlabelled specific probe (50‐fold); N: 400 nM SCAB_Lrp2 with nonspecific probe poly‐dIdC (50‐fold). (d) EMSAs of binding affinity of SCAB_Lrp2 to probe P_*txtR*_ in the presence of tryptophan.

Thaxtomin biosynthesis is activated by TxtR, the only CSR regulator in the *txt* cluster (Joshi et al. 2007; Yang, Tauschek, and Robins‐Browne 2011). To understand the regulatory role of SCAB_Lrp2 in thaxtomin A production, we assessed its impact on *txtR* expression using reverse transcription‐quantitative PCR (RT‐qPCR). The results showed that transcript levels of *txtR* in Δ*SCAB_Lrp2* increased by 9.4‐fold (*p* < 0.001) at 24 h and 8.9‐fold (*p* < 0.001) at 48 h compared to the wild‐type strain 87.22 (Figure 3a).

To determine whether SCAB_Lrp2 directly regulates *txtR* transcription, EMSAs were conducted with the *txtR* promoter DNA incubated with SCAB_Lrp2. The results showed that SCAB_Lrp2 could bind to the *txtR* promoter, forming the SCAB_Lrp2‐P_*txtR*_ complex (Figure 3b). Using the site OP1 from the *SCAB_Lrp2* promoter as a reference, EMSAs with the mutated probe P_d*txtR*_ identified a highly similar 18 bp sequence (5′‐GGCCAACTGCCGGTCGAT‐3′) in the *txtR* promoter region (named as site OP2) (Figure 3c). More importantly, SCAB_Lrp2 only responded to tryptophan when binding to the *txtR* promoter (Figure 3d), consistent with its behaviour when binding to its own promoter (Figure 1d).

In conclusion, these findings suggest that SCAB_Lrp2 negatively regulates thaxtomin A production by directly repressing the expression of the CSR gene *txtR* in *S. scabies*.

### 2.4 Deletion of *SCAB_Lrp2* Results in Hypervirulence of *S. scabies*

**FIGURE 4:**
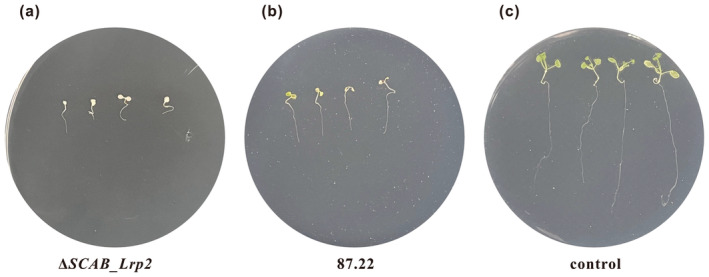
Deletion of *SCAB_Lrp2* results in hypervirulence of *Streptomyces scabies* 87.22. (a) Phenotype of *Arabidopsis thaliana* seedlings grown for 4 days in the presence of Δ*SCAB_Lrp2*. (b) Phenotype of *A. thaliana* seedlings grown for 4 days in the presence of 87.22. (c) Phenotype of *A. thaliana* seedlings grown for 4 days as the control.

To assess the effect of *SCAB_Lrp2* deletion on the virulence of *S. scabies*, we used *Arabidopsis thaliana* (ecotype Col‐0) seedlings, which are susceptible to thaxtomin A. Seedlings were treated with fermentation extracts from 87.22 and Δ*SCAB_Lrp2*, as previously described (Liu et al. 2023). After 4 days, seedlings treated with Δ*SCAB_Lrp2* extract (Figure 4a) displayed significantly greater inhibition of root and shoot growth compared to those treated with 87.22 extract (Figure 4b). The average length of seedlings inoculated with Δ*SCAB_Lrp2* was 1.12 cm (Figure 4a), representing a 52.7% decrease (*p* < 0.001) compared to the 1.71 cm length of seedlings inoculated with 87.22 (Figure 4b). These results demonstrate that *SCAB_Lrp2* deletion results in hypervirulence of *S. scabies*, which correlates with increased thaxtomin A production.

### 2.5 Tryptophan Strengthens SCAB_Lrp2‐Mediated Repression of Thaxtomin Biosynthesis via *txtR*

**FIGURE 5:**
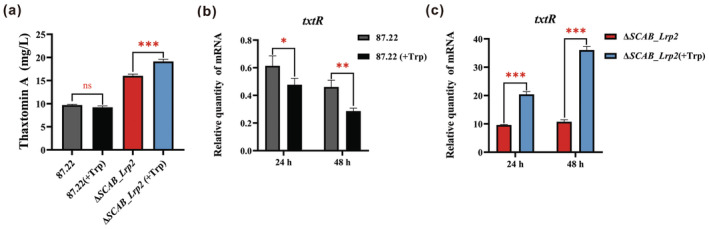
Tryptophan strengthens SACB_Lrp2‐mediated repression of thaxtomin biosynthesis via *txtR*. (a) Thaxtomin A production in 87.22 and Δ*SCAB_Lrp2* after adding 2.5 mM tryptophan through HPLC analysis. Mean values of three replicates are shown, with the standard deviation indicated by error bars (Student's *t* test; ns: not significant, ***p < 0.001). (b) Effects of adding 2.5 mM tryptophan on transcriptional levels of *txtR* in 87.22. Reverse transcription‐quantitative PCR (RT‐qPCR) was used to quantify the amounts of transcripts in 87.22 cultured for 24 and 48 h following tryptophan addition into oat bran broth (OBB). Mean values of three replicates are shown, with the standard deviation indicated by error bars (Student's *t* test; *p < 0.05, **p < 0.01). (c) Effects of adding 2.5 mM tryptophan on transcriptional levels of *txtR* in Δ*SCAB_Lrp2*. RT‐qPCR was used to quantify the amounts of transcripts in Δ*SCAB_Lrp2* cultured for 24 and 48 h following tryptophan addition into OBB medium. Mean values of three replicates are shown, with the standard deviation indicated by error bars (Student's *t* test; ***p < 0.001).

Tryptophan, a precursor of thaxtomin (Wang et al. 2020), was shown to act as a specific effector of SCAB_Lrp2 when binding to the *txtR* promoter (Figure 3d). To explore the impact of tryptophan on thaxtomin A production, 2.5 mM tryptophan was supplemented into the fermentation medium of *S. scabies* 87.22. Interestingly, this supplementation did not increase thaxtomin A production but instead caused a slight decrease (Figure 5a). RT‐qPCR analysis showed that *txtR* transcript levels decreased by 28.3% (*p* < 0.05) at 24 h and by 35.7% (*p* < 0.01) at 48 h following tryptophan addition in 87.22 (Figure 5b). In contrast, in the Δ*SCAB_Lrp2* strain, thaxtomin A production increased by 25.2% (*p* < 0.001) after tryptophan supplementation (Figure 5a). Furthermore, *txtR* transcript levels in Δ*SCAB_Lrp2* increased by 2.1‐fold (*p* < 0.001) at 24 h and by 3.4‐fold (*p* < 0.001) at 48 h following tryptophan addition (Figure 5c).

**FIGURE 6:**
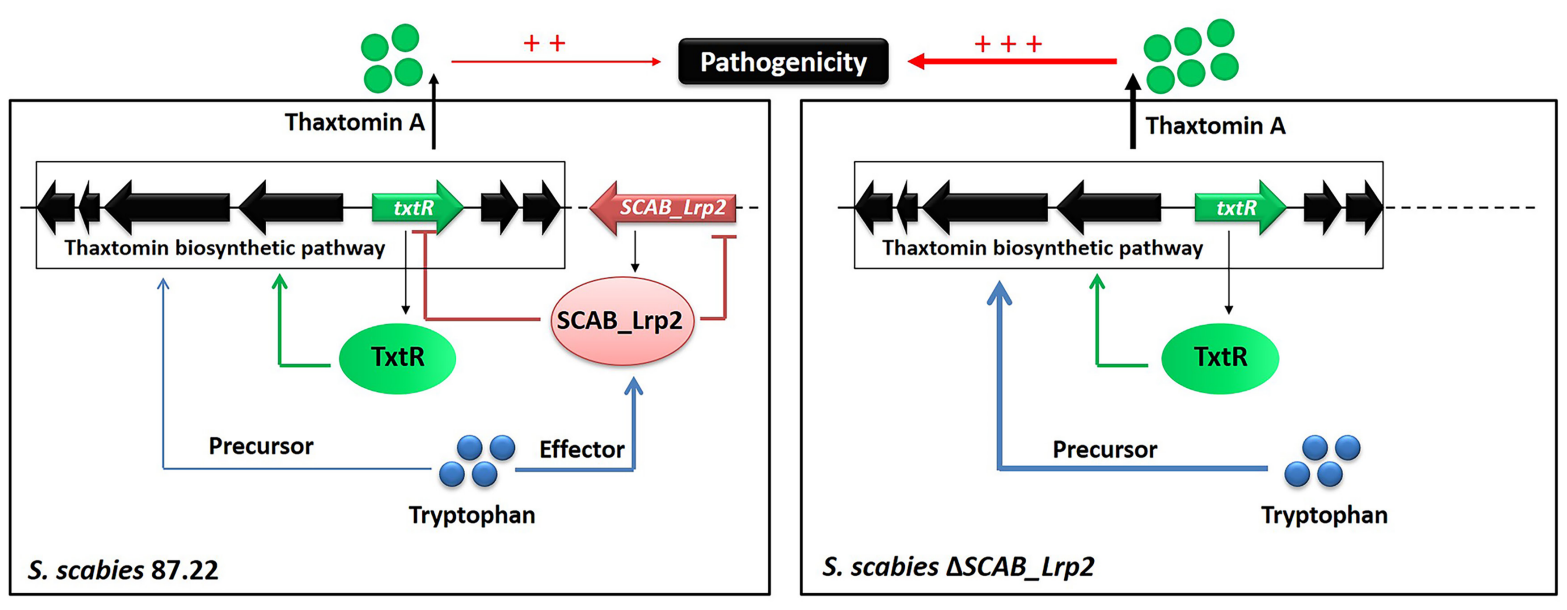
Proposed model of SCAB_Lrp2 responds to the precursor tryptophan and represses thaxtomin biosynthesis in *Streptomyces scabies* 87.22.

In summary, these findings suggest that tryptophan, as a precursor of thaxtomin, acts as an effector of SCAB_Lrp2, strengthening its repressive effect on thaxtomin biosynthesis through the CSR gene *txtR* (Figure 6).

## 3 Discussion

In *S. scabies*, our previous study has identified the Lrp family regulator SCAB_Lrp as a key activator of thaxtomin biosynthesis and morphological development (Liu et al. 2023). SCAB_Lrp positively regulates the transcription of the CSR gene *txtR* and the sporulation‐associated genes *amfC*, *whiB* and *ssgB* (Liu et al. 2023). This study highlighted a regulatory interaction between SCAB_Lrp and its analogue SCAB_Lrp2 in relation to their target genes. However, SCAB_Lrp2 does not respond to the amino acid effectors that influence SCAB_Lrp. In contrast to SCAB_Lrp, deletion of *SCAB_Lrp2* significantly increased thaxtomin A production and a hypervirulent phenotype developed in *S. scabies*. Moreover, tryptophan, a precursor of thaxtomin, acts as an effector for SCAB_Lrp2, strengthening its repression of thaxtomin biosynthesis via the CSR gene *txtR*. These observations provide new insights into the functional conservation and diversity among Lrp homologues in the biosynthesis of thaxtomin phytotoxins within pathogenic *Streptomyces*.

Recent advances in our laboratory have shed light on the molecular mechanisms of secondary metabolite biosynthesis regulated by Lrp family regulators, including SCAB_Lrp from *S. scabies* (Liu et al. 2023), SACE_Lrp from *Saccharopolyspora erythraea* (Liu et al. 2021, 2017a), and others across various *Streptomyces* species (Liu et al. 2019, 2017b; Xu et al. 2020). Sequence alignment reveals that SCAB_Lrp2 shares 32% identity with SCAB_Lrp, 30% identity with SACE_5717, and the lowest identity with the other proteins, suggesting varied regulatory functions of Lrp homologues among actinomycetes.

Several studies have extensively documented the direct regulatory capacities of Lrp regulators on their genes. EMSA results confirmed the ability of SCAB_Lrp2 to regulate its own gene's transcription by showing a distinct band shift when incubated with its promoter (Figure 1c). RT‐qPCR assays in Δ*SCAB_Lrp2* revealed a significant increase in *SCAB_Lrp2* transcript levels compared to the parent strain 87.22 (Figure S4), indicating its role as a repressor of its own gene.

Interestingly, while certain Lrp proteins such as SLCG_Lrp (Xu et al. 2020), SCO3361 (Liu et al. 2017b), and SACE_Lrp (Liu et al. 2017a), exhibit bidirectional responses to their effectors, altering their protein–DNA binding affinity, SACE_5717 (Liu et al. 2019) and SCAB_Lrp (Liu et al. 2023) show unidirectional responses. The presence of methionine and phenylalanine enhances the DNA‐binding affinity of SCAB_Lrp, leading to new protein–DNA complex (Liu et al. 2023). In contrast, SCAB_Lrp2 responds uniquely to tryptophan, improving its DNA‐binding capability without forming a new protein–DNA complex (Figures 1f and 3d). These findings highlight the intricate mechanisms by which Lrp proteins interact with different amino acid effectors.

## 4 Experimental Procedures

### 4.1 Strains and Growth Conditions

Table S1 presents the list of bacterial strains and plasmids used in this study. *S. scabies* and its derivatives were cultivated at 28°C on soy flour mannitol (SFM) solid medium for sporulation and phenotypic observation. Tryptic soy broth (TSB) was used for mycelial seed cultures (Liu et al. 2023), while thaxtomins were produced in OBB as previously described (Francis et al. 2015). *E. coli* strains were grown at 37°C in Luria Bertani (LB) medium. *E. coli* DH5α was used as the host for routine molecular cloning, and intergeneric conjugation was used to transfer DNA into *S. scabies* 87.22 (wild‐type strain) using *E. coli* ET12567 (pUZ8002). Protein overproduction was performed in *E. coli* BL21 (DE3). Antibiotic concentrations followed previously established protocols (Liu et al. 2023).

### 4.2 Overproduction and Purification of SCAB_Lrp2

Table S2 lists the primers used in this study. Genomic DNA extracted from *S. scabies* 87.22 was used to amplify *SCAB_Lrp2* (*SCAB_75421*) with primers 75421‐28a‐F/R (Table S2). The PCR product was digested with HindIII/NdeI and inserted into plasmid pET28a, generating pET*SCAB_Lrp2*. Subsequently, pET*SCAB_Lrp2* was introduced into *E. coli* BL21 (DE3) for SCAB_Lrp2 overexpression, which was induced by adding 0.5 mM isopropyl β‐d‐1‐thiogalactopyranoside (IPTG) for 20 h at 16°C. Cells were collected, lysed in a lysis buffer (20 mM Tris–HCl, 500 mM NaCl, 5 mM imidazole), and centrifuged after sonication. Next, recombinant His_6_‐tagged SCAB_Lrp2 and SCAB_Lrp (Liu et al. 2023) were purified using a Ni‐NTA spin column (TransGen Biotech), the His_6_‐tag was removed using a thrombin cleavage kit (Abcam). Purified proteins were analysed by SDS‐PAGE, and their concentrations were determined using a Bradford kit (Sangon Biotech).

### 4.3 EMSA Assays

Promoter regions of target genes were amplified by PCR from 87.22 genomic DNA using biotin‐labelled primers (Table S2). Purified PCR products were quantified and used in EMSA assays following the methods as described (Hellman and Fried 2007), using a chemiluminescent EMSA kit (Beyotime Biotechnology). The 20 μL binding reaction included 50 ng of labelled probe, varying amounts of purified SCAB_Lrp2, and 2 μL of binding buffer (Liu et al. 2023). Each probe was incubated separately with SCAB_Lrp2 following outlined methods (Liu et al. 2017a). To test competitive inhibition, 2500 ng of unlabelled probe was added to the reaction.

To identify the DNA‐binding site for SCAB_Lrp2, HindIII, NdeI and EcoRI sites were introduced into probe P_*75421*_ to replace the site OP1 sequence, generating the mutated probe P_d*75421*_. The same method was used to generate the mutated probe P_d*txtR*_. The SCAB_Lrp2 protein and probe P_*75421*_ were also incubated with various amino acids to investigate potential effectors, following the method described (Liu et al. 2019).

### 4.4 Gene Deletion

PCR was performed using specified primer pairs (Table S2) to amplify two 1500‐bp DNA fragments flanking the *SCAB_Lrp2* gene from the genomic DNA of strain 87.22. The amplified products were digested with HindIII/XbaI and KpnI/EcoRI and ligated into the corresponding sites of pUCTSR (Han et al. 2011) to generate plasmid pUCΔ*SCAB_Lrp2*. Subsequently, pUCΔ*SCAB_Lrp2* was transferred into *E. coli* ET12567 (pUZ8002) and then introduced into *S. scabies* 87.22 via intergeneric conjugation. The *tsr* replaced a 303‐bp fragment of *SCAB_Lrp2* through homologous chromosomal recombination. PCR analysis confirmed the deletion mutant Δ*SCAB_Lrp2* using primers 75421‐CF/R (Table S2).

### 4.5 Gene Complementation and Overexpression

For gene complementation of Δ*SCAB_Lrp2*, a 453‐bp fragment of the *SCAB_Lrp2* gene was amplified by PCR from the genomic DNA of strain 87.22 using primers 75421‐CF/R (Table S2). This amplified fragment was inserted into the NdeI/XbaI sites of plasmid pIB139 (Wilkinson et al. 2002), creating plasmid pIB*SCAB_Lrp2*. Both pIB139 and pIB*SCAB_Lrp2* were transferred into *E. coli* ET12567 (pUZ8002), following by intergenic conjugation with Δ*SCAB_Lrp2*, generating strains Δ*SCAB_Lrp2*/pIB139 and Δ*SCAB_Lrp2*/*SCAB_Lrp2*. Confirmation of these strains was done via PCR using primers apr‐TF/R (Table S2).

Additionally, wild‐type strain 87.22 was conjugated with pIB*SCAB_Lrp2* and pIB139 to create the overexpression strain 87.22/*SCAB_Lrp2* and control strain 87.22/pIB139. PCR confirmation was conducted via PCR using primers apr‐TF/R (Table S2).

### 4.6 Determination of Thaxtomin A Production

Quantified spores of *S. scabies* 87.22 and its derivatives were inoculated into TSB for seed culture, following the procedure outlined (Liu et al. 2023). Five millilitres of seed culture was transferred into 50 mL of OBB and incubated at 28°C with shaking at 220 rpm for 7 days. Thaxtomin A extraction and analysis were performed using an HPLC system with a Wondasil C18 Superb column (4.6 × 150 mm, 5 μm; GC Sciences), following the method outlined (Liu et al. 2023). Thaxtomin A levels were quantified by generating a standard curve with an authentic thaxtomin A standard (AbMole BioScience) and measuring absorption at 380 nm.

### 4.7 Cell Growth Measurement

To assess the effect of *SCAB_Lrp2* deletion on cell growth, the biomass of *S. scabies* 87.22 and mutant Δ*SCAB_Lrp2* was measured using the method described (Liu et al. 2023). Both strains were cultivated as described for thaxtomin A production. Culture samples were collected daily (1 mL), centrifuged, and the cell pellets were washed and dried for biomass determination, as previously described (Liu et al. 2017a).

### 4.8 Virulence Bioassays

To examine the impact of *SCAB_Lrp2* deletion on the pathogenic traits of *S. scabies*, virulence assays were performed on *A. thaliana* Col‐0 seedlings, as previously described (Francis et al. 2015). Seedlings were treated with 200 μL of fermentation extract from either wild‐type 87.22 or mutant Δ*SCAB_Lrp2* as outlined (Francis et al. 2015; Jourdan et al. 2016). Plates were incubated at 21°C ± 2°C with a 12‐h photoperiod for 3–4 days.

### 4.9 RNA Extraction and RT‐qPCR Assay

Total RNA was extracted from *S. scabies* 87.22 and Δ*SCAB_Lrp2* using TransZol reagent (TransGen Biotech). Both strains were cultured in OBB for 24 and 48 h. RNA (500 ng) was treated with 4× gDNA wiper Mix (Vazyme Biotech) before reverse transcription using a cDNA synthesis kit (Vazyme Biotech). qPCR was conducted using the Applied Biosystems QuantStudio 6 Flex system and 2 × AceQ Universal SYBR qPCR Master Mix* (Vazyme Biotech), following the manufacturer's instructions (Vazyme Biotech). The 16S rRNA gene from *S. scabies* was used as the internal reference. Primer sequences for RT‐qPCR are listed in Table S2.

## Conflicts of Interest

The authors declare no conflicts of interest.

## Supporting information


**Figure S1.** Sequence alignment of SCAB_Lrp2 and SCAB_Lrp. The black boxes represent the identical amino acid residues, while the grey boxes represent the similar amino acid residues.

**Figure S2.** Phenylalanine or methionine was not the effector of SCAB_Lrp2. (a) Electrophoretic mobility shift assays (EMSAs) of binding affinity of SCAB_Lrp2 to probe P_*75421*_ in presence of phenylalanine. (b) EMSAs of binding affinity of SCAB_Lrp2 to probe P_*75421*_ in presence of methionine.

**Figure S3.** Electrophoretic mobility shift assays of binding affinity of SCAB_Lrp2 to probe P_*75421*_ present in different protein amino acids of 20 mM. (a) The amount of SCAB_Lrp2 used was 100 nM. (b) The amount of SCAB_Lrp2 used was 500 nM. C, the control with no amino acid added. Trp, Tyr, Asp, Cys and Glu are dissolved in 0.1 M NaOH, and the other amino acids are dissolved in water.

**Figure S4.** The transcriptional level of *SCAB_Lrp2* in 87.22 and Δ*SCAB_Lrp2* by reverse transcription‐quantitative PCR analysis. Mean values of three replicates are shown, with the standard deviation indicated by error bars (Student’s *t* test; ***p < 0.001).

**Table S1.** Strains and plasmids used in this study.

**Table S2.** Primers used in this study.

## Data Availability

All data generated or analysed during this study are available from the corresponding author on reasonable request.
