# Supplementary figures and images for: Lrp Family Regulator SCAB_Lrp2 Responds to the Precursor Tryptophan and Represses the Thaxtomin Biosynthesis in Streptomyces scabies
Source: Mol Plant Pathol. 2024 Dec 1;25(12):e70036. doi: 10.1111/mpp.70036 (PMC11609053; doi:10.1111/mpp.70036)

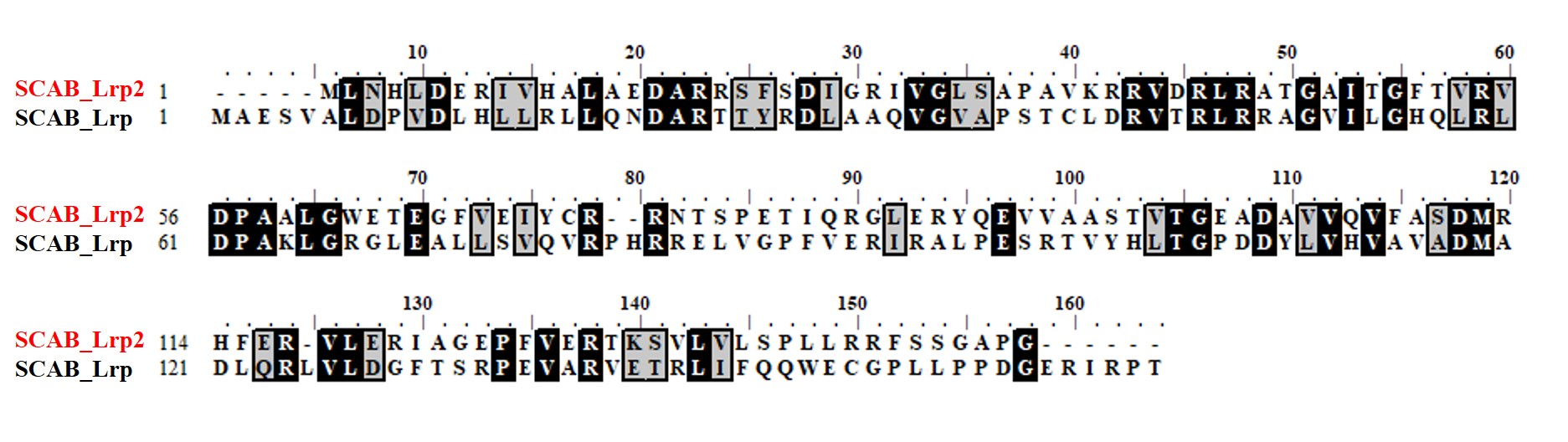

Supplement: Supplementary file 1 — Figure S1. Sequence alignment of SCAB_Lrp2 and SCAB_Lrp. The black boxes represent the identical amino acid residues, while the grey boxes represent the similar amino acid residues. [file MPP-25-e70036-s005.jpg]

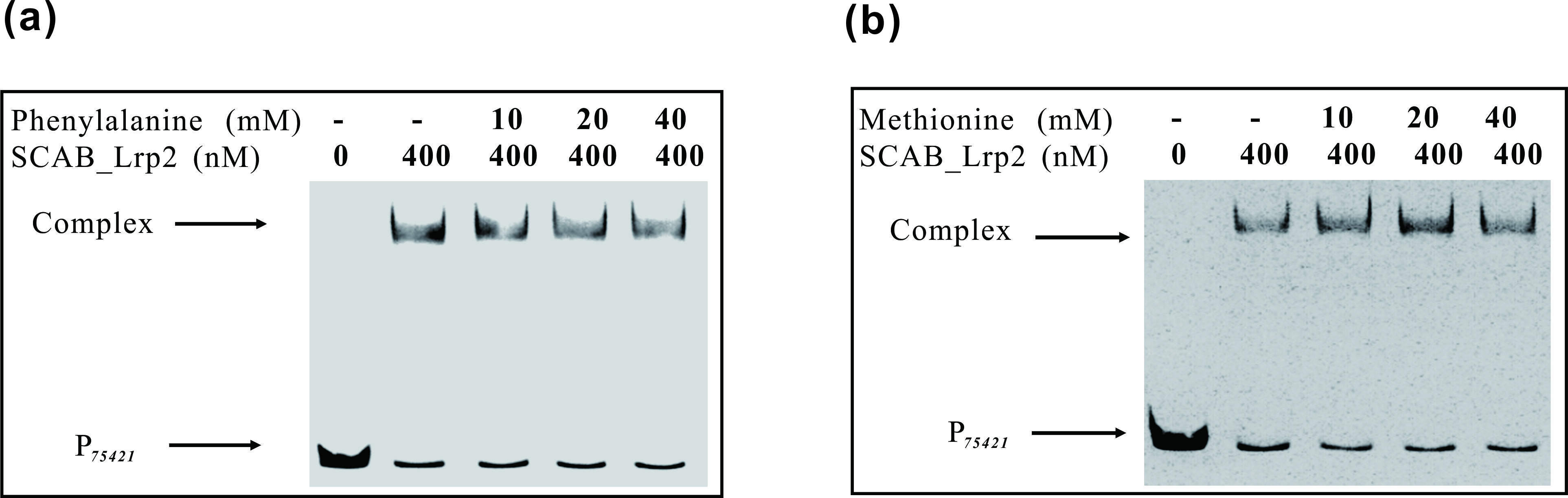

Supplement: Supplementary file 2 — Figure S2. Phenylalanine or methionine was not the effector of SCAB_Lrp2. (a) Electrophoretic mobility shift assays (EMSAs) of binding affinity of SCAB_Lrp2 to probe P 75421 in presence of phenylalanine. (b) EMSAs of binding affinity of SCAB_Lrp2 to probe P 75421 in presence of methionine. [file MPP-25-e70036-s006.jpg]

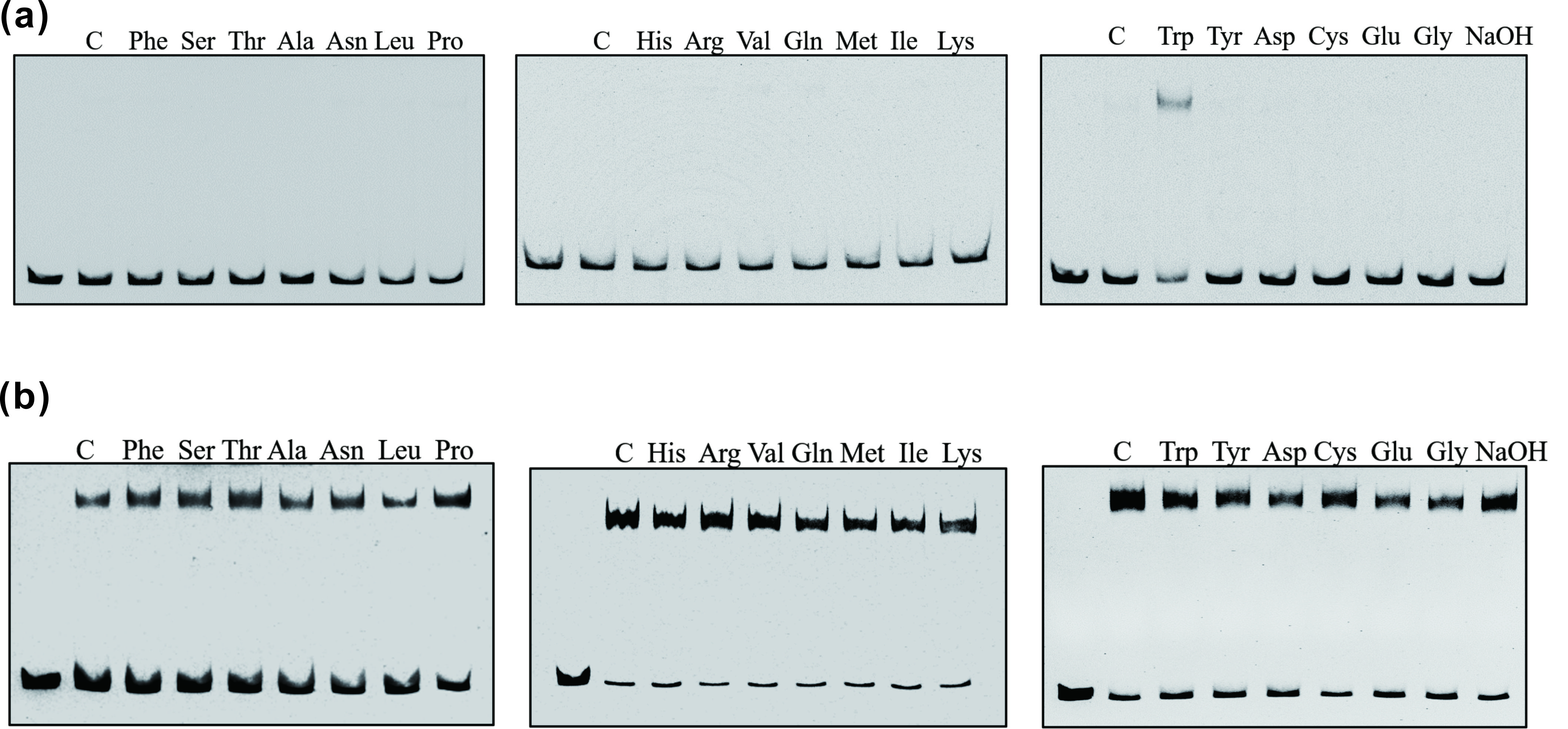

Supplement: Supplementary file 3 — Figure S3. Electrophoretic mobility shift assays of binding affinity of SCAB_Lrp2 to probe P 75421 present in different protein amino acids of 20 mM. (a) The amount of SCAB_Lrp2 used was 100 nM. (b) The amount of SCAB_Lrp2 used was 500 nM. C, the control with no amino acid added. Trp, Tyr, Asp, Cys and Glu are dissolved in 0.1 M NaOH, and the other amino acids are dissolved in water. [file MPP-25-e70036-s002.jpg]

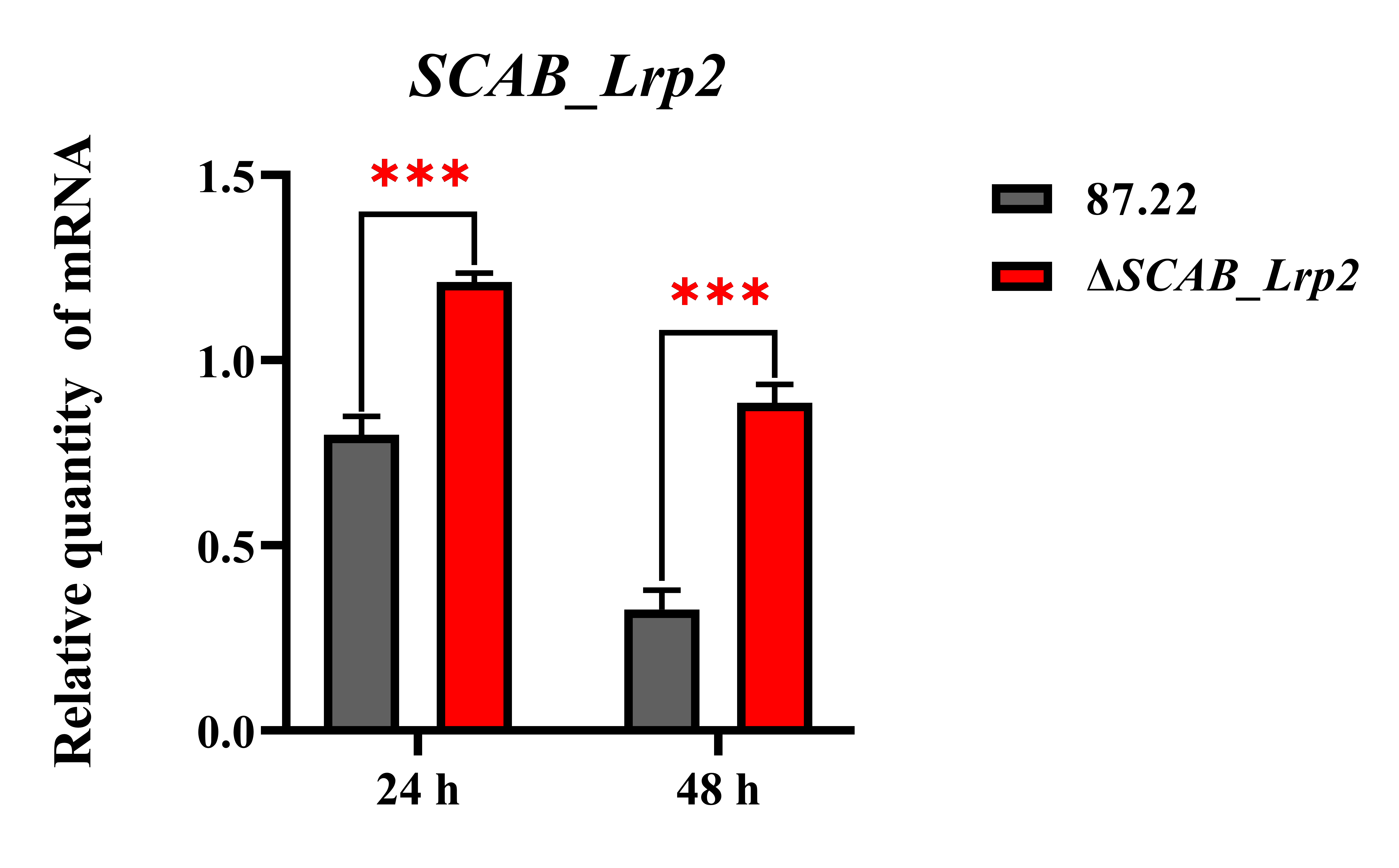

Supplement: Supplementary file 4 — Figure S4. The transcriptional level of SCAB_Lrp2 in 87.22 and ΔSCAB_Lrp2 by reverse transcription‐quantitative PCR analysis. Mean values of three replicates are shown, with the standard deviation indicated by error bars (Student’s t test; ***p < 0.001). [file MPP-25-e70036-s003.png]
