# Supplementary material for: Lrp Family Regulator SCAB_Lrp2 Responds to the Precursor Tryptophan and Represses the Thaxtomin Biosynthesis in Streptomyces scabies
Source: Mol Plant Pathol. 2024 Dec 1;25(12):e70036. doi: 10.1111/mpp.70036 (PMC11609053; doi:10.1111/mpp.70036)
Supplement: Supplementary file 5 — Table S1. Strains and plasmids used in this study. [file MPP-25-e70036-s001.docx]

**Table S1** Strains and plasmids used in this study

| **Strains or plasmids** | **Description** | **Sources** |
| --- | --- | --- |
| S. scabies strains | | |
| 87.22 | Wild-type strain | (Loria et al., 1995) |
| ΔSCAB_Lrp2 | 87.22 with SCAB_Lrp2 deleted | This study |
| ΔSCAB_Lrp2/pIB139 | Δ*SCAB_*Lrp2 carrying pIB139 | This study |
| ΔSCAB_Lrp2/SCAB_Lrp2 | Δ*SCAB_*Lrp2 carrying pIBSCAB_Lrp2 | This study |
| 87.22/pIB139 | 87.22 carrying pIB139 | This study |
| 87.22/SCAB_Lrp2 | 87.22 carrying pIBSCAB_Lrp2 | This study |
| ***E. coli*** strains | | |
| DH5α | F *recA lacZ*M15 | Invitrogen |
| BL21(DE3) | F-*ompThsd*SB (*rB*^-^*mB*^-^) *gal dcm* (DE3) | Novagen |
| ET12567(pUZ8002) | *recF dam- dcm- hsdS cat Km* | (Kieser et al., 2000) |
| **Plasmids** | | |
| pUCTSR | pUC18 derivative containing a 1.36-kb fragment of a thiostrepton resistance cassette in the *Bam*HI/*Sma*I sites | (Han et al. 2011) |
| pUCΔ*SCAB_*Lrp2 | pUCTSR derivative for *SCAB_*Lrp2 deletion | This study |
| pIB139 | *aac(3)IV*, P*ermE**origin1 | (Kieser et al., 2000) |
| pIB*SCAB_*Lrp2 | pIB139 derivative for expression of *SCAB_*Lrp2 | This study |
| pET28a | *kan*, P*_T7_*, His-tag | Novagen |
| pET*SCAB_*Lrp2 | pET28a derivative carrying *SCAB_*Lrp2 | This study |
| pET-*SCAB_*Lrp | pET28a derivative carrying *SCAB_*Lrp | (Liu et al., 2023) |

**References**

Loria, R., Bukhalid, R. A., Creath, R. A., Leiner, R. H., Olivier, M. and Steffens, J. C. (1995) Differential production of thaxtomins by pathogenic *Streptomyces* species *in vitro*. *Phytopathology*, 85, 537-541.

Kieser, T., Bibb, M. J., Buttner, M. J., Chater, K. F., Hopwood, D. A. (2000) Practical *Streptomyces* Genetics. John Innes Foundation, Norwich.

Han, S., Song, P., Ren, T., Huang, X., Cao, C. and Zhang, B. (2011) Identification of SACE_7040, a member of TetR family related to the morphological differentiation of *Saccharopolyspora erythraea*. *Current Microbiology,* 63, 121-125.

Liu, J., Wang, Y., He, H., Dong, S., Tang, L., Wang, Y.*,* et al. (2017a) The leucine-responsive regulatory protein SCAB_Lrp modulates thaxtomin biosynthesis, pathogenicity, and morphological development in *Streptomyces scabies*. *Molecular Plant Pathology,* 24, 167-178.
