# Supplementary material for: Lrp Family Regulator SCAB_Lrp2 Responds to the Precursor Tryptophan and Represses the Thaxtomin Biosynthesis in Streptomyces scabies
Source: Mol Plant Pathol. 2024 Dec 1;25(12):e70036. doi: 10.1111/mpp.70036 (PMC11609053; doi:10.1111/mpp.70036)
Supplement: Supplementary file 6 — Table S2. Primers used in this study. [file MPP-25-e70036-s004.docx]

**Table S2** Primers used in this study

| Name | Sequence (5′-3′) (restriction site underlined) | Use |
| --- | --- | --- |
| 75421-up-F | AAAAAGCTTACTGTGGCAGCTGACGATGAT (*Hin*dIII) | PCR amplification and identification for *SCAB_Lrp2* deletion |
| 75421-up-R | AAATCTAGAGCCGATGTCGGAGAAGGAACG (*Xba*I) |  |
| 75421-down-F | AAAGGTACCTTCGTCGAGCGGACCAAGTCG (*Kpn*I) |  |
| 75421-down-R | AAAGAATTCGGGGGCGAGAAGGCCCTGCTG (*Eco*RI) |  |
| 75421-CF | AAACATATGGTGCTGAACCATCTCGACGAA (*Nde*I) |  |
| 75421-CR | AAATCTAGATCAGCCGGGCGCCCCGGAGGA (*Xba*I) |  |
| apr-TF | ggAgTgCATATggTgCAATACgAATggCgA |  |
| apr-TR | CTCAAAgCTTCAgCCAATCgACTggCgAgC |  |
| 75421-28a-F | AAACATATGGTGCTGAACCATCTCGACGAA (*Nde*I) | Expression of SCAB_Lrp2 |
| 75421-28a-R | AAAAAGCTTTCAGCCGGGCGCCCCGGAGGA (*Hin*dIII) |  |
| 75421-E-F | GTCGTCTTCGGCGTCTGGATG | EMSA analysis |
| 75421-E-R | TCGTCGAGATGGTTCAGCACT |  |
| d75421-E-R | TGCGGGTGGATCACTTCTCTGGAGAATTCCATATGAAGCTT  GGGGGAGCCG |  |
| txtR-E-F | CGATTGACCTGGTAGTTGAAA |  |
| txtR-E-R | AAGGCAGTACCTTGCGCTCGG |  |
| dtxtR-E-R | AACAGTTCAGAACAGGCCACGGCGAGATCCTCCGCGAATTC  CATATGAAGCTTTCACCCGCTT |  |
| 16sRNA-RT-F | ATCCGATGAGTGTGGAAAGC | qRT-PCR analysis |
| 16sRNA-RT-R | GTGCAATATTCCCCACTGCT |  |
| txtR-RT-F | GGATGCGATCCACTTCTGAT |  |
| txtR-RT-R | CGCACCGATATGTTGTGTTC |  |
| 75421-RT-F | TCGGACATGCGGCACTTCG |  |
| 75421-RT-R | CAGCGGCGAGAGCACCAG |  |

F stands for forward primer, and R stands for reverse primer.
